# Supplementary material for: Can Circulating MicroRNAs, Cytokines, and Adipokines Help to Differentiate Psoriatic Arthritis from Erosive Osteoarthritis of the Hand? A Case–Control Study
Source: Int J Mol Sci. 2025 May 12;26(10):4621. doi: 10.3390/ijms26104621 (PMC12111288; doi:10.3390/ijms26104621)
Supplement: Supplementary file 1 [file ijms-26-04621-s001.zip › Figure S1.pdf]

miR-21 (RE)	0.582	0.475	0.423	0.384	0.376	-0.103	0.128	0.332	0.477	0.280	0.326	0.135	0.470	0.500	0.196	0.108	0.244	0.148	0.001	-0.098	0.056
0.582	miR-140 (RE)	0.497	0.475	0.474	0.322	0.147	0.251	0.344	0.403	0.416	0.482	0.222	0.507	0.501	0.322	0.305	0.147	0.235	0.106	0.121	0.199
0.475	0.497	miR-146a (RE)	0.185	0.418	0.470	0.429	0.209	0.293	0.288	0.441	0.499	0.186	0.400	0.374	0.252	0.402	0.328	0.298	0.337	0.237	0.280
0.423	0.475	0.185	miR-155 (RE)	0.574	0.340	-0.159	0.215	0.366	0.439	0.352	0.246	0.308	0.607	0.733	0.357	0.158	0.200	0.407	-0.124	-0.096	-0.020
0.384	0.474	0.418	0.574	miR-181b (RE)	0.406	0.188	0.322	0.428	0.413	0.507	0.462	0.336	0.644	0.722	0.399	0.368	0.312	0.576	0.194	0.080	0.300
0.376	0.322	0.470	0.340	0.406	miR-223 (RE)	0.266	0.194	0.263	0.236	0.383	0.359	0.238	0.454	0.525	0.302	0.425	0.294	0.467	0.271	0.037	0.267
-0.103	0.147	0.429	-0.159	0.188	0.266	miR-let-7e (RE)	0.052	0.048	-0.073	0.320	0.366	0.188	0.144	0.010	0.148	0.527	0.278	0.383	0.512	0.442	0.495
0.128	0.251	0.209	0.215	0.322	0.194	0.052	IL-1β (RE)	0.378	0.148	0.353	0.450	0.133	0.387	0.395	0.389	0.335	0.047	0.240	0.338	0.232	0.290
0.332	0.344	0.293	0.366	0.428	0.263	0.048	0.378	IL-6 (RE)	0.443	0.645	0.561	0.200	0.532	0.501	0.356	0.371	0.245	0.238	0.288	0.162	0.301
0.477	0.403	0.288	0.439	0.413	0.236	-0.073	0.148	0.443	TNF-α (RE)	0.417	0.428	0.085	0.431	0.479	0.307	0.223	0.178	0.123	0.073	0.040	0.136
0.280	0.416	0.441	0.352	0.507	0.383	0.320	0.353	0.645	0.417	IL-17a (RE)	0.666	0.270	0.509	0.574	0.488	0.556	0.298	0.448	0.417	0.334	0.459
0.326	0.482	0.499	0.246	0.462	0.359	0.366	0.450	0.561	0.428	0.666	IL-23a (RE)	0.180	0.600	0.511	0.529	0.557	0.241	0.407	0.550	0.428	0.538
0.135	0.222	0.186	0.308	0.336	0.238	0.188	0.133	0.200	0.085	0.270	0.180	Serum IL1-β (pg/mL)	0.263	0.305	0.174	0.248	0.146	0.187	0.077	-0.038	0.070
0.470	0.507	0.400	0.607	0.644	0.454	0.144	0.387	0.532	0.431	0.509	0.600	0.263	Serum IL-6 (pg/mL)	0.838	0.586	0.441	0.309	0.581	0.326	0.175	0.364
0.500	0.501	0.374	0.733	0.722	0.525	0.010	0.395	0.501	0.479	0.574	0.511	0.305	0.838	Serum TNF-α (pg/mL)	0.581	0.414	0.324	0.621	0.217	0.043	0.256
0.196	0.322	0.252	0.357	0.399	0.302	0.148	0.389	0.356	0.307	0.488	0.529	0.174	0.586	0.581	Serum IL-17a (pg/mL)	0.454	0.198	0.475	0.345	0.310	0.428
0.108	0.305	0.402	0.158	0.368	0.425	0.527	0.335	0.371	0.223	0.556	0.557	0.248	0.441	0.414	0.454	Serum IL-23a (pg/mL)	0.219	0.503	0.564	0.468	0.626
0.244	0.147	0.328	0.200	0.312	0.294	0.278	0.047	0.245	0.178	0.298	0.241	0.146	0.309	0.324	0.198	0.219	Serum adiponectin (μg/mL)	0.307	0.145	0.001	0.231
0.148	0.235	0.298	0.407	0.576	0.467	0.383	0.240	0.238	0.123	0.448	0.407	0.187	0.581	0.621	0.475	0.503	0.307	Serum chemerin (pg/mL)	0.440	0.268	0.492
0.001	0.106	0.337	-0.124	0.194	0.271	0.512	0.338	0.288	0.073	0.417	0.550	0.077	0.326	0.217	0.345	0.564	0.145	0.440	Serum leptin (pg/mL)	0.514	0.648
-0.098	0.121	0.237	-0.096	0.080	0.037	0.442	0.232	0.162	0.040	0.334	0.428	-0.038	0.175	0.043	0.310	0.468	0.001	0.268	0.514	Serum visfatin (ng/mL)	0.479
0.056	0.199	0.280	-0.020	0.300	0.267	0.495	0.290	0.301	0.136	0.459	0.538	0.070	0.364	0.256	0.428	0.626	0.231	0.492	0.648	0.479	Serum resistin (pg/mL)

Figure S1. Association between miRNAs, cytokines gene expression and serum cytokines and adipokines as detected by Spearman's rank correlation coefficient.
