# Supplementary material for: Can Circulating MicroRNAs, Cytokines, and Adipokines Help to Differentiate Psoriatic Arthritis from Erosive Osteoarthritis of the Hand? A Case–Control Study
Source: Int J Mol Sci. 2025 May 12;26(10):4621. doi: 10.3390/ijms26104621 (PMC12111288; doi:10.3390/ijms26104621)
Supplement: Supplementary file 1 [file ijms-26-04621-s001.zip › Table S1.pdf]

Table S1. Bivariable logistic regression models used to evaluate the ability of serum cytokines and adipokines to discriminate EHOA from PsA after correction for a known or potential confounder (sex, age, disease duration, BMI, tender joints and lnCRP).

	M1	M2	M3	M4	M5	M6	M7	M8
miR-155 (RE)	2.16***	2.14***	1.93***	2.19***	2.15***	2.56***	2.00***	
	[1.35,2.97]	[1.32,2.96]	[1.04,2.81]	[1.35,3.03]	[1.34,2.96]	[1.60,3.52]	[0.96,3.04]	
Male sex		1.06						
		[-0.17,2.29]						
Age (years)			0.15***					
			[0.06,0.24]					
Disease (months)				0.01				
				[-0.00,0.03]				
BMI (kg/m^2)					-0.02			
					[-0.17,0.13]			
Tender joints (number)						-0.22**		
						[-0.37,-0.08]		
LnCRP + 0.01							-2.59***	-2.89***
							[-3.91,-1.28]	[-4.05,-1.72]
Constant	-4.44***	-5.15***	-13.44***	-5.62***	-3.85	-3.54***	-5.48***	-1.71***
	[-6.15,-2.73]	[-7.19,-3.11]	[-19.54,-7.35]	[-7.92,-3.31]	[-8.17,0.47]	[-5.33,-1.75]	[-7.86,-3.10]	[-2.53,-0.89]
Observations	100	100	100	100	100	100	100	100
AIC	89	88	77	88	91	80	58	78
BIC	94	96	85	95	99	88	66	83
ROC -AUC	0.89	0.90	0.92	0.89	0.89	0.91	0.95	0.90
Nagelkerke R^2	0.55	0.58	0.65	0.58	0.55	0.63	0.77	0.64

95% confidence intervals in brackets

* $p < 0.05$, ** $p < 0.01$, *** $p < 0.001$

	M1	M2	M3	M4	M5	M6	M7	M8
IL-23A (RE)	-1.21***	-1.15***	-1.11***	-1.17***	-1.19***	-1.18***	-1.62***	
	[-1.76,-0.66]	[-1.69,-0.60]	[-1.73,-0.49]	[-1.73,-0.62]	[-1.75,-0.64]	[-1.73,-0.64]	[-2.52,-0.72]	
Male sex		0.94						
		[-0.03,1.92]						
Age (years)			0.16***					
			[0.08,0.24]					
Disease (months)				0.01				
				[-0.00,0.02]				
BMI (kg/m^2)					-0.06			
					[-0.20,0.09]			
Tender joints (number)						-0.11		
						[-0.22,0.00]		
LnCRP + 0.01							-3.08***	-2.89***
							[-4.38,-1.78]	[-4.05,-1.72]
Constant	2.39***	1.64*	-8.20**	1.69*	3.80*	3.14***	1.45	-1.71***
	[1.24,3.54]	[0.28,3.00]	[-13.37,-3.03]	[0.18,3.20]	[0.04,7.56]	[1.71,4.57]	[-0.30,3.20]	[-2.53,-0.89]
Observations	100	100	100	100	100	100	100	100
AIC	117	116	95	117	119	115	62	78
BIC	122	123	102	125	126	123	70	83
ROC -AUC	0.79	0.80	0.87	0.80	0.78	0.80	0.95	0.90
Nagelkerke R^2	0.30	0.34	0.53	0.32	0.31	0.34	0.75	0.64

95% confidence intervals in brackets
* $p < 0.05$, ** $p < 0.01$, *** $p < 0.001$

	M1	M2	M3	M4	M5	M6	M7	M8
IL-17A (RE)	-1.03***	-1.04***	-0.96**	-1.04***	-1.02***	-1.01***	-1.04*	
	[-1.62,-0.45]	[-1.64,-0.44]	[-1.61,-0.31]	[-1.63,-0.45]	[-1.61,-0.43]	[-1.60,-0.41]	[-1.95,-0.14]	
Male sex		1.17*						
		[0.22,2.12]						
Age (years)			0.18***					
			[0.10,0.26]					
Disease (months)				0.01*				
				[0.00,0.02]				
BMI (kg/m^2)					-0.06			
					[-0.18,0.06]			
Tender joints (number)						-0.09		
						[-0.20,0.01]		
LnCRP + 0.01							-2.86***	-2.89***
							[-4.08,-1.65]	[-4.05,-1.72]
Constant	1.76**	0.97	-9.85***	0.87	3.22	2.42***	0.09	-1.71***
	[0.69,2.83]	[-0.27,2.21]	[-15.05,-4.66]	[-0.47,2.21]	[-0.01,6.46]	[1.10,3.74]	[-1.55,1.72]	[-2.53,-0.89]
Observations	100	100	100	100	100	100	100	100
AIC	128	124	100	126	129	127	74	78
BIC	133	132	108	134	137	134	81	83
ROC -AUC	0.74	0.76	0.86	0.77	0.74	0.75	0.92	0.90
Nagelkerke R^2	0.18	0.25	0.48	0.22	0.19	0.22	0.68	0.64

95% confidence intervals in brackets
* $p < 0.05$, ** $p < 0.01$, *** $p < 0.001$

	M1	M2	M3	M4	M5	M6	M7	M8
Serum TNF-α (pg/mL)	0.18*** [0.09,0.27]	0.20*** [0.10,0.31]	0.18*** [0.08,0.27]	0.19*** [0.10,0.29]	0.18*** [0.10,0.27]	0.18*** [0.10,0.27]	0.29* [0.01,0.58]	
Male sex		-1.42 [-3.84,1.00]						
Age (years)			0.14 [-0.00,0.29]					
Disease (months)				-0.01 [-0.03,0.01]				
BMI (kg/m^2)					0.05 [-0.19,0.30]			
Tender joints (number)						0.07 [-0.19,0.32]		
LnCRP + 0.01							-5.91 [-11.95,0.13]	-2.89*** [-4.05,-1.72]
Constant	-28.72*** [-42.49,-14.95]	-31.07*** [-46.76,-15.39]	-37.03*** [-56.99,-17.07]	-29.58*** [-44.20,-14.95]	-30.25*** [-46.17,-14.33]	-29.33*** [-43.25,-15.40]	-49.92* [-98.36,-1.47]	-1.71*** [-2.53,-0.89]
Observations	100	100	100	100	100	100	100	100
AIC	34	35	32	36	36	36	18	78
BIC	39	43	40	43	44	44	25	83
ROC -AUC	0.98	0.99	0.99	0.99	0.98	0.99	1.00	0.90
Nagelkerke R^2	0.88	0.89	0.90	0.89	0.88	0.88	0.96	0.64

95% confidence intervals in brackets

* $p < 0.05$, ** $p < 0.01$, *** $p < 0.001$

	M1	M2	M3	M4	M5	M6	M7	M8
Serum leptin (pg/mL)	-0.00*** [-0.01,-0.00]	-0.00*** [-0.01,-0.00]	-0.00*** [-0.01,-0.00]	-0.00*** [-0.01,-0.00]	-0.00*** [-0.01,-0.00]	-0.00*** [-0.01,-0.00]	-0.00*** [-0.01,-0.00]	
Male sex		1.39* [0.08,2.70]						
Age (years)			0.23*** [0.10,0.35]					
Disease (months)				0.01 [-0.00,0.03]				
BMI (kg/m^2)					-0.12 [-0.32,0.09]			
Tender joints (number)						-0.14 [-0.30,0.02]		
LnCRP + 0.01							-2.64*** [-4.02,-1.27]	-2.89*** [-4.05,-1.72]
Constant	8.78*** [5.39,12.17]	8.19*** [4.68,11.71]	-4.57 [-11.87,2.74]	7.78*** [4.33,11.23]	11.90*** [5.04,18.77]	10.14*** [6.00,14.28]	7.62** [3.00,12.25]	-1.71*** [-2.53,-0.89]
Observations	100	100	100	100	100	100	100	100
AIC	81	78	59	79	82	80	49	78
BIC	86	86	67	87	89	88	57	83
ROC -AUC	0.91	0.92	0.96	0.92	0.91	0.91	0.97	0.90
Nagelkerke R^2	0.61	0.65	0.77	0.64	0.62	0.64	0.82	0.64

95% confidence intervals in brackets

* $p < 0.05$, ** $p < 0.01$, *** $p < 0.001$

	M1	M2	M3	M4	M5	M6	M7	M8
Serum visfatin (ng/mL)	-2.72*** [-3.77,-1.68]	-2.67*** [-3.72,-1.62]	-2.86*** [-4.09,-1.63]	-2.87*** [-3.99,-1.75]	-2.77*** [-3.83,-1.70]	-2.65*** [-3.70,-1.61]	-3.03*** [-4.64,-1.42]	
Male sex		0.96 [-0.22,2.13]						
Age (years)			0.18*** [0.08,0.29]					
Disease (months)				0.01* [0.00,0.03]				
BMI (kg/m^2)					-0.10 [-0.26,0.07]			
Tender joints (number)						-0.05 [-0.18,0.08]		
LnCRP + 0.01							-2.72*** [-4.05,-1.39]	-2.89*** [-4.05,-1.72]
Constant	6.29*** [3.90,8.68]	5.53*** [3.04,8.02]	-5.37 [-11.99,1.25]	5.36*** [2.85,7.87]	8.74*** [3.71,13.77]	6.51*** [4.04,8.98]	5.31** [1.87,8.75]	-1.71*** [-2.53,-0.89]
Observations	100	100	100	100	100	100	100	100
AIC	86	85	68	83	86	87	51	78
BIC	91	93	75	91	94	95	59	83
ROC -AUC	0.89	0.89	0.93	0.90	0.89	0.89	0.97	0.90
Nagelkerke R^2	0.58	0.60	0.72	0.61	0.59	0.58	0.81	0.64

95% confidence intervals in brackets
* $p < 0.05$, ** $p < 0.01$, *** $p < 0.001$
