# Supplementary material for: Can Circulating MicroRNAs, Cytokines, and Adipokines Help to Differentiate Psoriatic Arthritis from Erosive Osteoarthritis of the Hand? A Case–Control Study
Source: Int J Mol Sci. 2025 May 12;26(10):4621. doi: 10.3390/ijms26104621 (PMC12111288; doi:10.3390/ijms26104621)
Supplement: Supplementary file 1 [file ijms-26-04621-s001.zip › Table S2.pdf]

Table S2. List of primers analyzed by RT-qPCR

miRNA Genes	Cat. No. (Qiagen)
miR-21	MS00009079
miR-140	MS00003318
miR-146a	MS00003535
miR-155	MS00008778
miR-181b	MS00006692
miR-223	MS00003871
SNORD-25	MS00014007
Target Genes	Cat. No. (Qiagen)
IL-1β	QT00021385
IL-6	QT00083720
IL-17a	QT00009233
IL-23a	QT00088721
TNF-a	QT00029162
ACTB	QT00095431

Abbreviations: miRNA, microRNA; SNORD-25, Small Nucleolar RNA, C/D Box 25; IL,interleukin; TNF, tumor necrosis factor; ACTB, actin beta.
